# Supplementary material for: Simultaneous and independent topological control of identical microparticles in non-periodic energy landscapes
Source: Nat Commun. 2023 Nov 18;14:7517. doi: 10.1038/s41467-023-43390-0 (PMC10657436; doi:10.1038/s41467-023-43390-0)
Supplement: Supplementary file 1 — Supplementary Information [file 41467_2023_43390_MOESM1_ESM.pdf]

Supplementary Information
Simultaneous and independent topological control of identical microparticles in non-periodic energy landscapes

Nex C. X. Stuhlmüller,[1] Farzaneh Farrokhzad,[2] Piotr Kuświk,[3] Feliks Stobiecki,[3] Maciej Urbaniak,[3] Sapida Akhundzada,[4] Arno Ehresmann,[4] Thomas M. Fischer,[2] and Daniel de las Heras[1, *]

[1] *Theoretische Physik II, Physikalisches Institut, Universität Bayreuth, D-95440 Bayreuth, Germany*
[2] *Experimatalphysik X, Physikalisches Institut, Universität Bayreuth, D-95440 Bayreuth, Germany*
[3] *Institute of Molecular Physics, Polish Academy of Sciences, 60-179 Poznań, Poland*
[4] *Institute of Physics and Center for Interdisciplinary Nanostructure Science and Technology (CINSaT), University of Kassel, D-34132 Kassel, Germany*

(Dated: November 7, 2023)

SUPPLEMENTARY NOTE 1
SIMPLE PATTERNS AND SIMPLE LOOPS

We summarize here the topological transport control of isotropic magnetic colloidal particles above periodic magnetic patterns. A sketch of the system and the different types of periodic patterns is shown in Supplemental Fig. 1. Detailed theoretical and experimental studies can be found in Refs. [1–3]. For a given orientation of the external magnetic field, there is in general at least one minimum of the magnetic potential per Wigner-Seitz cell. During a modulation loop, the changes in the orientation of $\mathbf{H}_{\text{ext}}$ are slow enough such that the colloidal particles can follow a minimum of the magnetic potential at every time. In this sense the colloidal motion is adiabatic except if the minimum that transports a particles disappears (e.g. due to the annihilation with a saddle point). In such cases, the colloidal particle performs a ratchet motion towards a minimum nearby.

To control the colloidal transport we therefore need to understand the stationary points of the magnetic potential. The position of the minima in action space depends on the orientation of $\mathbf{H}_{\text{ext}}$ and on the symmetry of the pattern. By analysing the eigenvectors and the eigenvalues of the Hessian matrix of the magnetic potential, it turns out [1–3] that action space can be split into allowed and forbidden regions for the colloidal particles, see Supplemental Fig. 2. For each space point in an allowed region it is always possible to find an orientation of $\mathbf{H}_{\text{ext}}$ such that the magnetic potential is a minimum. Note also that a minimum of V_{mag} can be transformed into a maximum by simply inverting the external field since $V_{\text{mag}} \propto \mathbf{H}_{\text{p}} \cdot \mathbf{H}_{\text{ext}}$. Hence both minima and maxima of V_{mag} can be found in the allowed regions. For each space point in a forbidden region, there is an orientation of $\mathbf{H}_{\text{ext}}$ such that the magnetic potential is a saddle point, but never a minimum.

The boundary between the allowed and the forbidden regions are the fences. The location of the fences in both action space and control space depend on the symmetry of the pattern. In a square pattern, the fences in $\mathcal{C}$ are four equidistant points on the equator, see Supplemental Fig. 1(a). In hexagonal patterns however the fences are curves, the shape and the position of which vary with the symmetry phase ϕ, Supplemental Fig. 1(c). Crucially, in hexagonal patterns the fences of a given pattern and its corresponding inverse pattern (opposite magnetization) do not coincide in control space, cf. the top and the bottom patterns in Supplemental Fig. 1(c). As we discuss now, this means that the transport in a given pattern and its inverse pattern can be independently controlled with a single modulation loop.

The position of the fences is relevant to control the colloidal motion, which in action space occurs through the allowed regions. Two adjacent allowed regions are connected via points that we refer to as the gates, see Supplemental Fig. 2. To adiabatically transport a particle from one allowed region to an adjacent allowed region, we need to modulate $\mathbf{H}_{\text{ext}}$ in $\mathcal{C}$ such that a minimum of the potential crosses the gate that connects both regions. To induce transport between two consecutive Wigner-Seitz cells using closed modulation loops in $\mathcal{C}$, the loop in $\mathcal{C}$ needs to be such that the particle crosses two different gates once the loop returns to its initial position. In square patterns such loops are those that wind around the fence points [2, 3] in $\mathcal{C}$, see an example in Supplemental Fig. 2(a). In hexagonal patterns, the fences in $\mathcal{C}$ are curves made of twelve segments. Two fence segments in $\mathcal{C}$ meet at a bifurcation point. The loops that induce transport in hexagonal patterns are those that wind around at least three consecutive bifurcation points of the fences [1, 3] (enclosing therefore at least two consecutive fence segments). The bifurcation points are indicated

* delasheras.daniel@gmail.com; www.danieldelasheras.com

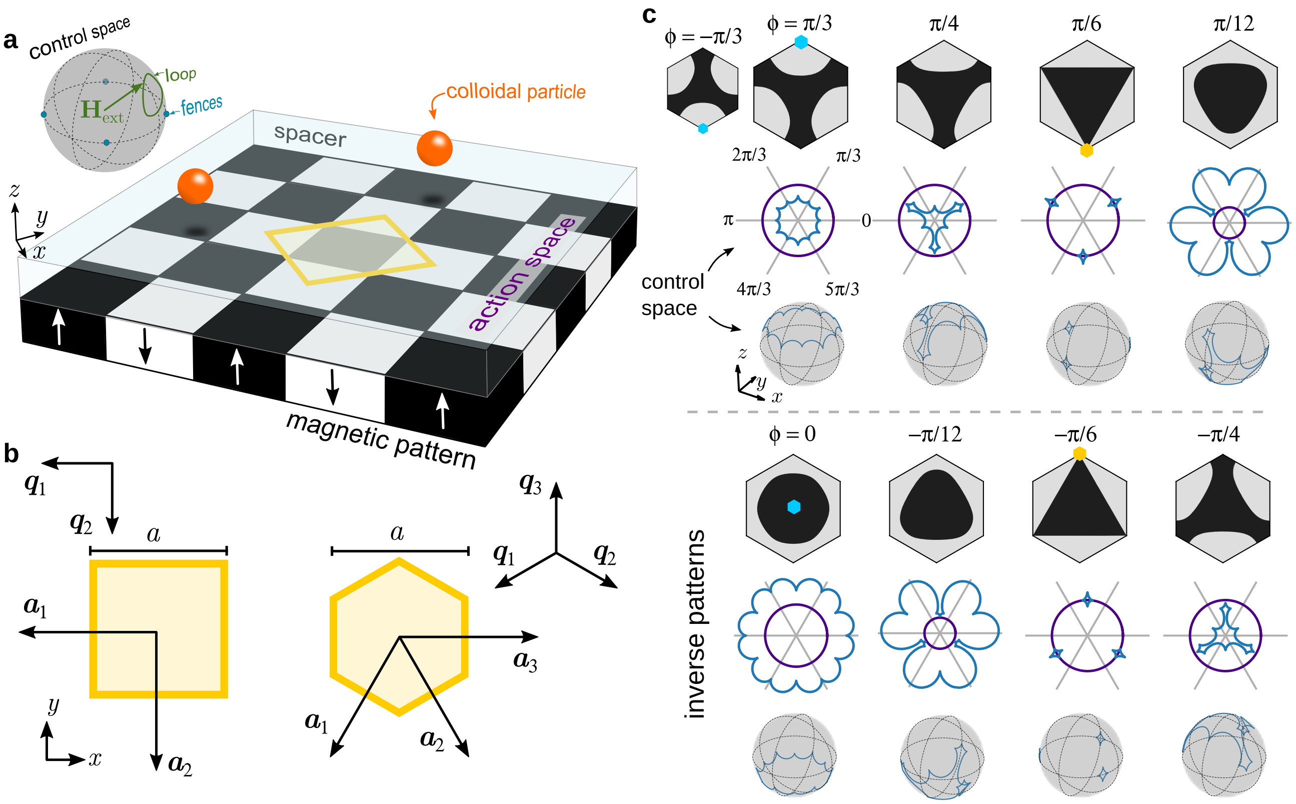

Supplementary Fig. 1. **Setup and magnetic patterns.** (a) Sketch of the system: a square magnetic pattern with domains of positive (black) and negative (white) magnetization parallel to the normal of the pattern. A Wigner-Seitz cell is highlighted in yellow. Identical colloidal particles are located above the pattern. A spacer restricts the particle motion to action space $\mathcal{A}$, a plane parallel to the pattern. An external magnetic field $\mathbf{H}_{\rm ext}$ spatially uniform (green arrow) drives the motion via closed loops (green loop) of its orientation in control space $\mathcal{C}$ (sphere). The fences in $\mathcal{C}$ are represented in blue. (b) Wigner-Seitz cells in square and hexagonal patterns. The lattice vectors $\mathbf{a}_i$ and the wave vectors $\mathbf{q}_i$ are also shown. The magnitude of the lattice vectors is a. In the experiments $a = 14\,\mu$m. (c) Magnetization of Wigner-Seitz cells and corresponding control spaces in a family of hexagonal patterns with varying symmetry phase ϕ, as indicated. The fences are represented in blue. The control space is represented via a sphere and also using a stereographic projection in which the equator is represented as a violet circle. The patterns in the bottom row have the inverse magnetization than those in the upper row and the unit cell is also shifted. The yellow and the blue hexagons indicate the position of points with S_6 and C_6 symmetry, respectively.

in Supplemental Fig. 2(b) and Supplemental Fig. 2(c) for patterns with C_6 and S_6 symmetries, respectively, together with illustrative examples of loops that induce transport.

The simplest but non-trivial modulation loops that induce net motion are those that transport the particles along the symmetry directions of the pattern. These are given by lattice vectors $\pm\mathbf{a}_i$ with $i = 1, ..., N$ and $N = 2$ ($N = 3$) in square (hexagonal) patterns, see Supplemental Fig. 1. Illustrative examples of such modulation loops are shown in Supplemental Fig. 2.

The transport in square patterns is always adiabatic, and reversing the modulation loop reverses also the direction of transport [2]. In contrast, in hexagonal patterns the transport can be either adiabatic or ratchet-like [1, 3]. In the latter case, reversing the loop does not always reverse the direction of the transport. However, the direction of the transport is in all cases deterministic and topologically protected.

The set of winding numbers of the modulation loop around the fences (square patterns) and around the bifurcation points (hexagonal patterns) is the topological invariant that protects the motion. Any two loops with the same set of winding numbers (topological invariant) will transport a particle in the same direction, even though the detailed trajectories depend of course on the particular shapes of the loops.

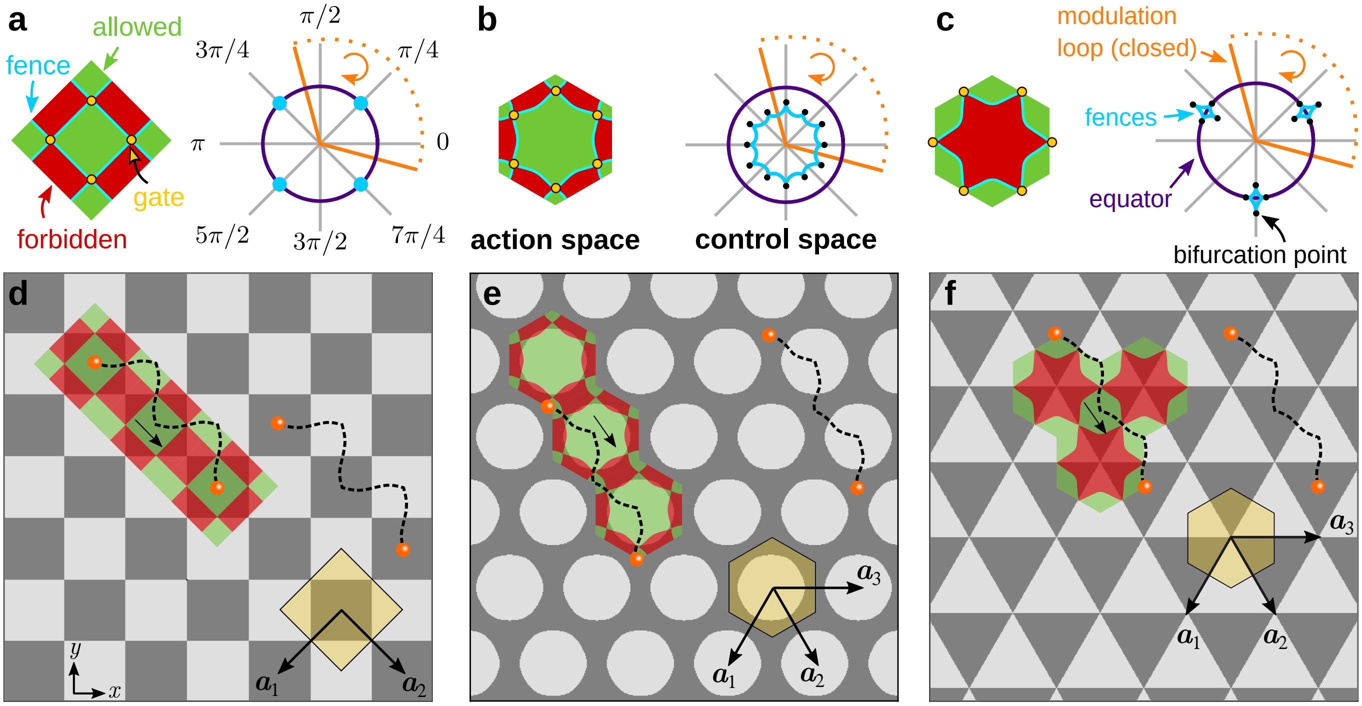

Supplementary Fig. 2. **Simple patterns and simple loops.** Action space and control space in square patterns (a) and hexagonal patterns with C_6 (b) and S_6 (c) symmetries. A unit cell illustrating the allowed (green) and forbidden (red) regions of action space, as well as the fences (blue lines) and the gates (yellow circles) is represented in each case. The control spaces (stereographic projections) show the equator (violet circle), the fences (blue), and a modulation loop (orange). The twelve bifurcation points in $\mathcal{C}$ of the fences of C_6 and S_6 patterns are also indicated with black circles. The modulation loop is the same in all cases and it is made of two segments of constant azimuthal angle joined at the north and the south poles of control space. The connection at the south pole (not visible due to the stereographic projection) is illustrated with a dotted orange segment. The loops wind anticlockwise, as indicated by the circular orange arrows. Magnetization in patterns with square (d), C_6 (e), and S_6 (f) symmetries. Black (white) regions are up (down) magnetized. The global phase is set to $\psi = \pi/4$ (d), $\psi = \pi/3$ (e), and $\psi = \pi/6$ (f). Black dashed lines are Brownian dynamics simulations of the trajectories of colloidal particles (orange circles) subjected to two consecutive modulation loops. The transport direction is indicated with black arrows. The trajectories go along the allowed regions only. A unit cell of each pattern with corresponding lattice vectors $\mathbf{a}_i$ is highlighted in yellow. For visualization purposes we have shifted the unit cells of the C_6 and the S_6 patterns with respect to those represented in Supplemental Fig. 1.

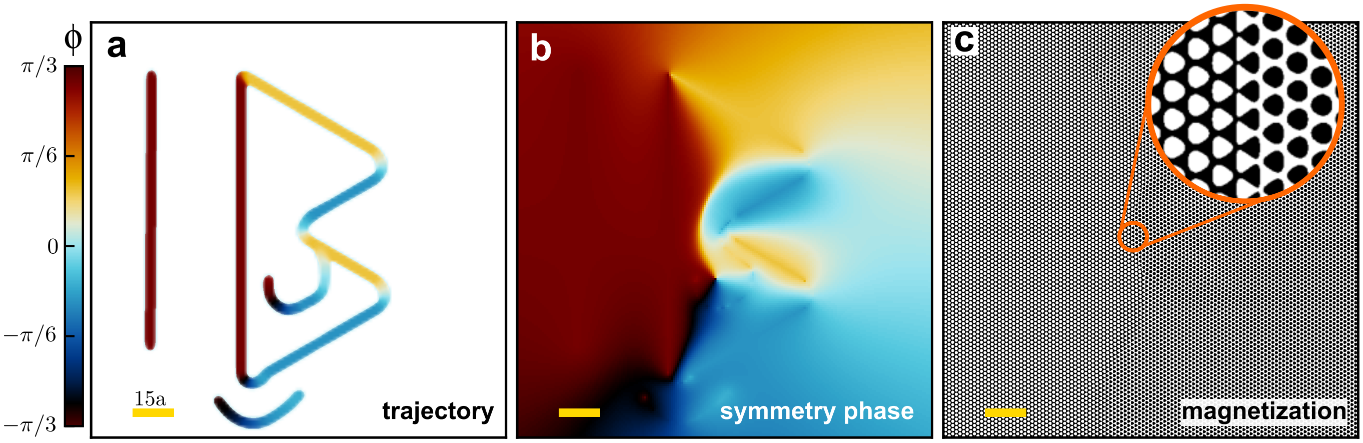

Supplementary Fig. 3. **Generation of symmetry phase modulated patterns.** (a) Trajectory drawn in Krita and colored according to the desired symmetry phase (color bar). The actual line is 1 pixel thick. Here we have made the trajectory thicker for visualization purposes. (b) Symmetry phase in the whole pattern calculated using the value of the symmetry phase along the trajectory. (c) Final magnetization of the pattern. The magnetization is positive in the black regions and negative in the white regions. The inset is a close view of a small region of the pattern, as indicated. Approximately the same region of the experimental pattern is highlighted in Fig. 4(c) of the main text. The length of the scale bars (yellow) is $15a$.

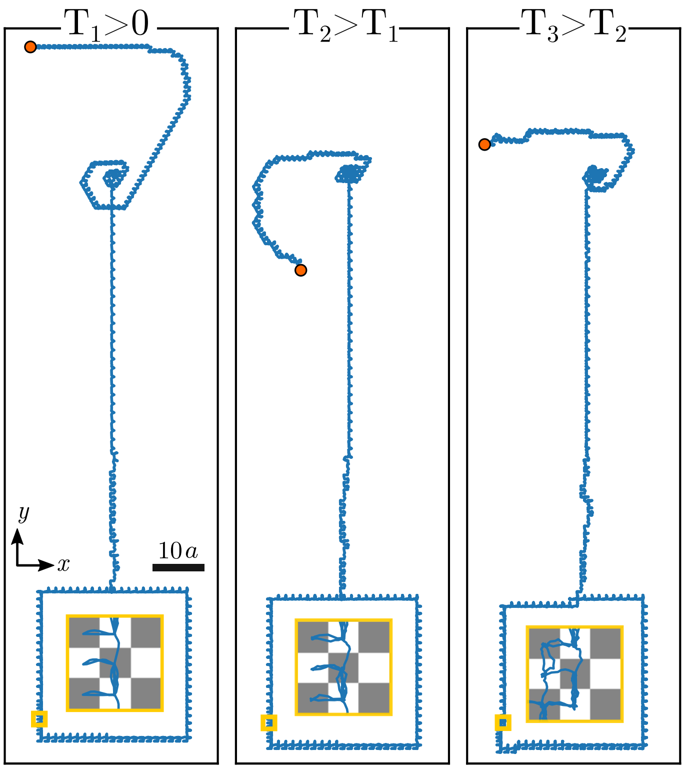

Supplementary Fig. 4. **Finite temperature effects.** Brownian dynamics simulations of colloidal particles moving above an inhomogeneous pattern at three different finite temperatures: $k_B T_1/\varepsilon \approx 3 \cdot 10^{-3}$, $k_B T_2/\varepsilon \approx 1 \cdot 10^{-2}$, and $k_B T_1/\varepsilon \approx 2 \cdot 10^{-2}$. The energy scale ϵ is the absolute value of the average external energy that a particle has when the external field points normal to the pattern. The particle trajectories are represented in blue (the starting point is indicated with an orange circle). The scale bar is $10a$. The pattern is made of two subpatterns: a top subpattern with a topological defect in the symmetry phase and a bottom subpattern with square symmetry. The insets are closed views of a small region (indicated by a yellow square) showing the trajectory (blue) and the magnetization of the pattern.

SUPPLEMENTARY REFERENCES

[1] J. Loehr, M. Loenne, A. Ernst, D. de las Heras, and T. M. Fischer, Topological protection of multiparticle dissipative transport, Nat. Commun. **7**, 11745 (2016).
[2] D. de las Heras, J. Loehr, M. Loenne, and T. M. Fischer, Topologically protected colloidal transport above a square magnetic lattice, New. J. Phys. **18**, 105009 (2016).
[3] J. Loehr, D. de las Heras, M. Loenne, J. Bugase, A. Jarosz, M. Urbaniak, F. Stobiecki, A. Tomita, R. Huhnstock, I. Koch, A. Ehresmann, D. Holzinger, and T. M. Fischer, Lattice symmetries and the topologically protected transport of colloidal particles, Soft Matter **13**, 5044 (2017).
