# Supplementary material for: Simultaneous and independent topological control of identical microparticles in non-periodic energy landscapes
Source: Nat Commun. 2023 Nov 18;14:7517. doi: 10.1038/s41467-023-43390-0 (PMC10657436; doi:10.1038/s41467-023-43390-0)
Supplement: Supplementary file 3 — Description of Additional Supplementary Files [file 41467_2023_43390_MOESM3_ESM.pdf]

Description of Additional Supplementary Files

Supplementary movie – Movie1.webm: A particle trap

Trajectories of colloidal particles above a pattern with a topological defect on the symmetry phase that acts as an attractor of particles.

Supplementary movie – Movie2.webm: Complex patterns and simple loops

Colloidal particles above a symmetry phase modulated pattern follow a predesigned trajectory with the shape of a B

Supplementary movie – Movie3.webm: The alphabet

Colloidal particles above rotated square patterns and subject to a complex loop follow trajectories writing the first 18 letters of the alphabet.

Supplementary movie – Movie4.webm: ABCD

A side-by-side comparison between experiments and simulations of colloidal particles above square patterns that differ in their global orientation.

Supplementary movie – Movie5.webm: Complex patterns and complex loops

Three colloidal particles at unknown positions are initialized using particle traps and from them force to follow complex trajectories.
